# Supplementary material for: Recipes for improper ferroelectricity in molecular perovskites
Source: Nat Commun. 2018 Jun 18;9:2380. doi: 10.1038/s41467-018-04764-x (PMC6006342; doi:10.1038/s41467-018-04764-x)
Supplement: Supplementary file 1 — Supplementary Information [file 41467_2018_4764_MOESM1_ESM.pdf]

Recipes for Improper Ferroelectricity in Molecular Perovskites

Boström et al.

Supplementary Information

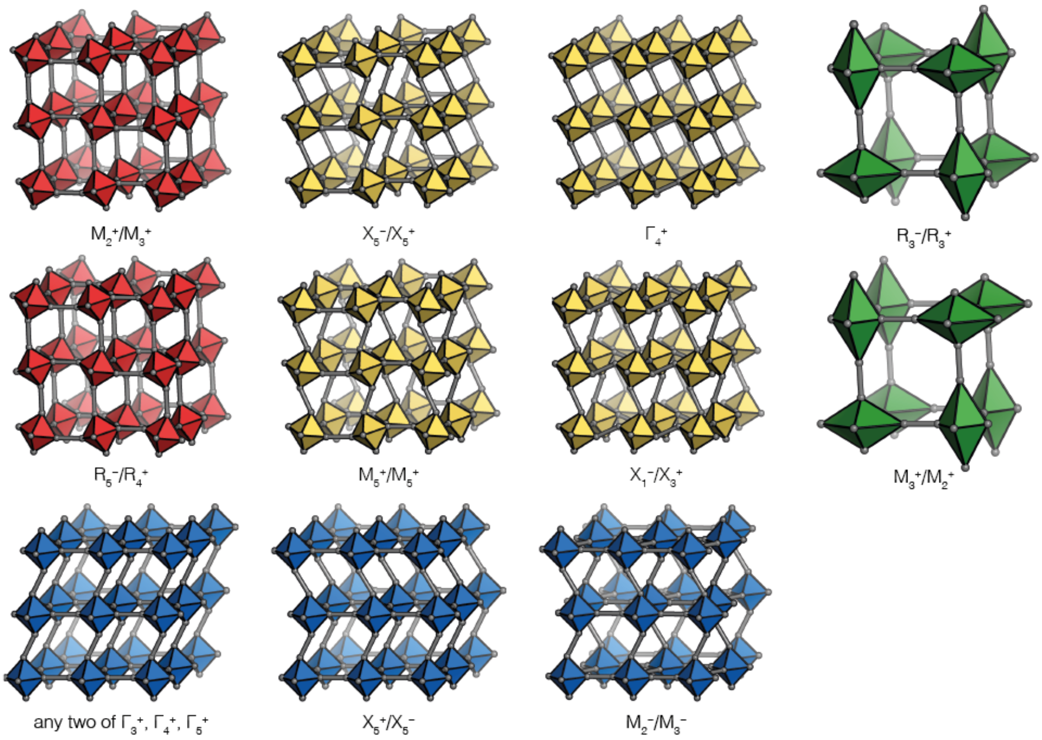

Supplementary Figure 1. Common structural distortions of the molecular perovskites. Conventional tilting is shown in red, unconventional tilting in yellow, columnar shifts in blue and Jahn-Teller distortions in green. The pictures are accompanied by the irrep in the setting with the A-site/B-site at the origin.

Supplementary Table 1. Conversion of irreps for the different settings of the perovskite unit cell.

Distortion	Origin at A	Origin at B
Symmetric strain	Γ_1^+	Γ_1^+
Tetragonal strain	Γ_3^+	Γ_3^+
Unconventional tilt	Γ_4^+	Γ_4^+
Orthorhombic strain	Γ_5^+	Γ_5^+
Acoustic polar mode	Γ_4^-	Γ_4^-
Octahedral deformation	Γ_5^-	Γ_5^-
Out-of-phase shift	X_1^+	X_3^-
Octahedral deformation	X_2^+	X_4^-
Columnar shift	X_5^+	X_5^-
Unconventional tilt	X_1^-	X_3^+
Octahedral deformation	X_2^-	X_4^+
Jahn-Teller distortion	X_3^-	X_1^+
Jahn-Teller distortion	X_4^-	X_2^+
Unconventional tilt	X_5^-	X_5^+
Octahedral deformation	M_1^+	M_4^+
Conventional tilt	M_2^+	M_3^+
Jahn-Teller distortion	M_3^+	M_2^+
Jahn-Teller distortion	M_4^+	M_1^+
Unconventional tilt	M_5^+	M_5^+
Octahedral deformation	M_1^-	M_4^-
Columnar shift	M_2^-	M_3^-
A-site displacement	M_3^-	M_2^-
Out-of-phase shift	M_5^-	M_5^-
Octahedral deformation	R_4^+	R_5^-
Out-of-phase shift	R_5^+	R_4^-
B-site cation order	R_2^-	R_1^+
Jahn-Teller distortion	R_3^-	R_3^+
Octahedral deformation	R_4^-	R_5^+
Conventional tilt	R_5^-	R_4^+

Supplementary Table 2. Third-order couplings with polarisation (Γ_4^-). The coupling of one irrep from each column in a given row will generate a polar distortion, provided that crystal momentum is conserved (*e.g* X_1^+[½,0,0] + X_3^-[½,0,0] but not X_1^+[½,0,0] + X_3^-[0,½,0]). Coupling schemes satisfying the restrictions for the conservation of parity and crystal moments NOT listed here may still give piezoelectric space groups.

$R^+_{4,5}$	$R^-_{1,2,3,4,5}$
$R^-_{4,5}$	$R^+_{1,2,3,4,5}$
M^+_5	$M^-_{1,2,3,4,5}$
M^-_5	$M^+_{1,2,3,4,5}$
$M^+_{1,2}$	$M^-_{3,4}$
$M^-_{1,2}$	$M^+_{3,4}$
X_5^+	$X^-_{1,2,3,4,5}$
X_5^-	$X^+_{1,2,3,4,5}$
$X^+_{1,2}$	$X^-_{3,4}$
$X^-_{1,2}$	$X^+_{3,4}$

Supplementary Table 3. Fourth-order couplings with polarisation (Γ_4^-). The coupling of one irrep from each column in a given row will generate a polar distortion, provided that crystal momentum is conserved (*e.g* X_1^+[0,½,0] + M_5^-[½,0,½] + R_2^+[½,½,½], but not X_1^+[0,½,0] + M_5^-[0,½,½] + R_2^+[½,½,½]). Coupling schemes satisfying the restrictions for the conservation of parity and crystal moments NOT listed here may still give piezoelectric space groups.

$X^+_{1,2}$	$M^+_{3,4,5}$	$R^-_{1,2,3,4,5}$
$X^+_{1,2}$	$M^-_{3,4,5}$	$R^+_{1,2,3,4,5}$
$X^-_{1,2}$	$M^+_{3,4,5}$	$R^+_{1,2,3,4,5}$
$X^-_{1,2}$	$M^-_{3,4,5}$	$R^-_{1,2,3,4,5}$
$X^+_{3,4}$	$M^+_{1,2,5}$	$R^-_{1,2,3,4,5}$
$X^+_{3,4}$	$M^-_{1,2,5}$	$R^+_{1,2,3,4,5}$
$X^-_{3,4}$	$M^+_{1,2,5}$	$R^+_{1,2,3,4,5}$
$X^-_{3,4}$	$M^-_{1,2,5}$	$R^-_{1,2,3,4,5}$
X_5^+	$M^+_{1,2,3,4,5}$	$R^-_{1,2,3,4,5}$
X_5^+	$M^-_{1,2,3,4,5}$	$R^+_{1,2,3,4,5}$
X_5^-	$M^+_{1,2,3,4,5}$	$R^+_{1,2,3,4,5}$
X_5^-	$M^-_{1,2,3,4,5}$	$R^-_{1,2,3,4,5}$
$X^+_{1,2,3,4,5}$	$M^+_{1,2,3,4,5}$	$R^-_{4,5}$
$X^+_{1,2,3,4,5}$	$M^-_{1,2,3,4,5}$	$R^+_{4,5}$
$X^-_{1,2,3,4,5}$	$M^+_{1,2,3,4,5}$	$R^+_{4,5}$
$X^-_{1,2,3,4,5}$	$M^-_{1,2,3,4,5}$	$R^-_{4,5}$
